# Supplementary material for: Dietary Macronutrient Intake and the Gut Microbiome in Adults Undergoing Bariatric Surgery for Obesity
Source: medRxiv. 2025 Oct 30:2025.10.28.25338397. Preprint. [Version 1] doi: 10.1101/2025.10.28.25338397 (PMC12636640; doi:10.1101/2025.10.28.25338397)
Supplement: 1 [file NIHPP2025.10.28.25338397V1-supplement-1.pdf]

medRxiv preprint doi: https://doi.org/10.1101/2025.10.28.25338397; this version posted October 30, 2025. The copyright holder for this preprint (which was not certified by peer review) is the author/funder, who has granted medRxiv a license to display the preprint in perpetuity.
It is made available under a CC-BY 4.0 International license .

Supplemental Table 1: Mean Daily Dietary Intake of Macronutrients and Dietary Quality Determined Before and After Bariatric Surgery

Overall Dietary Quality	Healthy Eating Index (HEI) 2015
Caloric Intake	Energy (kcal)
Indexes of Fat Intake	% Kcal as Fat (%)
	Fat (g)/1000 kcal
	% Calories from MUFA (%)
	% Calories from PUFA (%)
	% Calories from SFA (%)
	Polyunsaturated to Saturated Fat Ratio
	Omega-3 Fatty Acids (g)/1000 kcal
	Omega-6 Fatty Acids (g)/1000 kcal
Indexes of Protein Intake	% Kcal as Protein (%)
	Protein (g)/1000 kcal
Indexes of Carbohydrate Intake	% Kcal as Carbohydrate (%)
	Carbohydrate (g)/1000 kcal
	Glycemic Index (glucose reference)
	Glycemic Load (glucose reference)/1000 kcal
	Added Sugars (by Total Sugars) (g)/1000 kcal
Indexes of Grain Intake	Whole Grains (ounce equivalents)/1000 kcal
	Refined Grains (ounce equivalents)/1000 kcal
Indexes of Fiber Intake	Total Fiber (g)/1000 kcal
	Soluble Dietary Fiber (g)/1000 kcal
	Insoluble Dietary Fiber (g)/1000 kcal

MUFA = monounsaturated fatty acids; PUFA = polyunsaturated fatty acids; SFA = saturated fatty acids
